# Complementary and Alternative Medicine Use and Its Association with Health-Related Quality of Life in Patients Receiving Maintenance Hemodialysis: A Cross-Sectional Study

**DOI:** 10.3390/healthcare14213708

**Published:** 2026-11-01

**Authors:** Pei-Chun Hsiung, Zhi-Hao Chen, Wen-Chin Lee, Yung-Hsiang Chen, Yu-Chuen Huang, Ming-Yen Tsai

**Affiliations:** 1 Department of Chinese Medicine, Kaohsiung Chang Gung Memorial Hospital and Chang Gung University College of Medicine, Kaohsiung 833, Taiwan; bbbear777@cgmh.org.tw (P.-C.H.); chzhhao@cgmh.org.tw (Z.-H.C.); 2 Division of Nephrology, Department of Internal Medicine, Kaohsiung Chang Gung Memorial Hospital and Chang Gung University College of Medicine, Kaohsiung 833, Taiwan; leewc@cgmh.org.tw; 3 Graduate Institute of Integrated Medicine, College of Chinese Medicine, China Medical University, Taichung 404, Taiwan; yhchen@mail.cmu.edu.tw; 4 Department of Medical Research, China Medical University Hospital, Taichung 404, Taiwan; yuchuen@mail.cmu.edu.tw; 5 Office of Research & Development, Department of Psychology, College of Medical and Health Science, Asia University, Taichung 413, Taiwan; 6 Departments of Medicine, University of California, Irvine, Irvine, CA 92697, USA; 7 School of Chinese Medicine, College of Chinese Medicine, China Medical University, Taichung 404, Taiwan

**Keywords:** chronic kidney disease, hemodialysis, complementary and alternative medicine, health-related quality of life, cross-sectional study

## Abstract

**Highlights:**

**What are the main findings?**

• CAM use was reported by 55.8% of patients receiving maintenance hemodialysis.
• Female sex was independently associated with CAM use.
• CAM users had higher mental health and social support scores, but most HRQOL domains showed no significant differences.

**What are the implications of the main findings?**

• Routine, nonjudgmental assessment of CAM use may improve communication and help identify potential safety concerns in dialysis care.
• The observed HRQOL associations do not establish a direct benefit of CAM; longitudinal studies are needed to clarify causality.

**Abstract:**

**Objective:** Complementary and alternative medicine (CAM) use is common among patients receiving maintenance hemodialysis (MHD), but its associated factors, communication patterns, and relationship with health-related quality of life (HRQOL) remain unclear. **Methods:** This single-center cross-sectional study included 154 patients receiving MHD in southern Taiwan. We assessed CAM use, physician disclosure, and HRQOL (measured via the Kidney Disease Quality of Life Short-Form Survey (KDQOL-SF v1.3)). Between-group comparisons (with Bonferroni correction), multivariate logistic regression, and general linear modeling were applied for data analysis. **Results:** Overall, 86 patients (55.8%) reported CAM use, most commonly traditional herbal-based medicine (65.1%). Female sex was independently associated with CAM use (adjusted odds ratio [AOR] = 2.56, 95% confidence interval [CI]: 1.28–5.13; *p* = 0.008). Among CAM users, 66.3% discussed CAM use with physicians, while 33.7% did not. After adjusting for age, gender, duration of dialysis, and comorbidities, CAM users demonstrated significantly higher social support (mean difference [MD] = 9.06 ± 2.65; 95% CI: 3.82, 14.30; *p* = 0.001) and mental component summary scores (MD = 5.41 ± 1.68; 95% CI: 2.09, 8.73; *p* = 0.002) than non-users. Conversely, CAM users had slightly lower symptom/problem list scores (MD = −3.56 ± 1.58; 95% CI: −6.68, −0.44; *p* = 0.026). **Conclusions:** CAM use was common and associated with female sex, better mental well-being, and perceived social support. Routine, open, and nonjudgmental communication about CAM is warranted. Multicenter longitudinal studies are needed to clarify these associations and their implications for HRQOL and clinical outcomes.

## 1. Introduction

End-stage renal failure represents an irreversible loss of kidney function. When renal performance declines to less than 15% of normal capacity, patients are considered to have reached end-stage renal disease (ESRD) [1]. At this stage, either kidney transplantation or dialysis becomes essential for sustaining life. In 2022, approximately 97,000 patients in Taiwan received hemodialysis [2], corresponding to an incidence rate of 536 per million members of the population [3]. According to the World Health Organization (WHO), the global incidence of ESRD continues to increase by approximately 5–10% annually [4].

Hemodialysis is a complex and demanding treatment that imposes multiple physical, psychological, and social burdens on patients. Individuals undergoing hemodialysis must regularly visit healthcare centers, spend prolonged hours receiving treatment, adhere strictly to medications, and maintain rigid dietary and fluid restrictions, often resulting in major lifestyle adjustments. These challenges may contribute to psychological distress, including anxiety, depression, and hopelessness [5], as well as physical symptoms such as fatigue, pain, muscle cramps, hypotension, insomnia, and pruritus [6]. Consequently, the health-related quality of life (HRQOL) of hemodialysis patients is significantly reduced compared to healthy individuals [7]. ESRD and its treatments are closely associated with diminished HRQOL, increased physical and mental health problems, and limited social participation [7,8]. Poor HRQOL and functional decline have been linked to higher mortality risks among dialysis patients [9], while comorbidities such as depression and anemia further worsen outcomes [10,11].

To improve life quality and mitigate the adverse effects of the disease and its treatment, many patients with chronic conditions like ESRD turn to complementary and alternative medicine (CAM) [12]. CAM refers to a broad range of therapeutic approaches used alongside conventional medicine to promote general well-being or manage specific conditions [13]. According to the WHO, over thousands of types of CAMs are practiced worldwide, encompassing natural products (e.g., herbal remedies, vitamins, probiotics), mind–body practices, acupuncture, massage, and other forms of traditional medicine. A systematic review of 41 global studies reported that 43% of adult hemodialysis patients used some form of CAM to alleviate disease-related symptoms and treatment side effects [14]. Common motivations for CAM use among patients with chronic kidney disease (CKD) include managing comorbidities and complications, improving overall health, and supporting renal recovery [14]. Enhancing HRQOL is another key factor influencing CAM use [15,16]. In a cross-sectional study, Dehghan et al. found that CAM users had higher overall HRQOL scores than non-users [17]. Moreover, hemodialysis patients who engaged in mind–body therapies reported significant improvements in physical functioning, pain reduction, general health, and social interaction compared with non-users [17].

Taiwan has one of the highest ESRD prevalence rates globally, with approximately one in every 650 individuals receiving dialysis [3]. Our previous report showed that the use of CAM among maintenance hemodialysis (MHD) patients in Taiwan was common [18]. However, the extent of CAM use and its associated factors among patients with MHD and their potential effects on life quality remain insufficiently explored. Therefore, this secondary analysis of the previously described cohort study aimed to investigate the predictors of CAM use and the impact on patients’ HRQOL and gain a comprehensive understanding of patients’ perspectives of CAM use among Taiwanese adults undergoing hemodialysis.

## 2. Materials and Methods

### 2.1. Study Design and Populations

From August 2022 to July 2024, all patients with ESRD undergoing MHD for more than 3 months at the dialysis center of Kaohsiung Chang Gung Memorial Hospital, an academic medical center in Kaohsiung, Taiwan, were assessed for eligibility. Patients were approached at their bedside during dialysis sessions and invited to participate. Exclusion criteria included age under 20 years, inability to speak Mandarin or Hokkien, and a diagnosis of dementia or psychiatric illness. The study was reported in accordance with the STROBE statement (checklist items provided in Appendix A) and approved by the Institutional Review Board of the Chang Gung Medical Foundation (approval No. 202200422B0). Written informed consent was obtained by a GCP-certified research assistant, and questionnaire responses were pseudonymized, stored securely, and handled confidentially.

Based on Jeon et al.’s study [19], who reported a 24.6% prevalence of CAM use among hemodialysis patients, using G*power 3.1.9.2 (Heinrich Heine University Düsseldorf, Düsseldorf, Germany), a sample size of 128 was calculated by setting the response distribution to 0.50, the error margin to 5%, and the confidence interval at 95%. The target sample was increased to 154 participants to reduce the nonresponse rate and improve reliability. A simple random sample was chosen from a registration list of all patients in our Dialysis Data Management Center through a random number generator.

### 2.2. Survey Instrument and Data Collection

Data were collected using a structured, three-part questionnaire covering demographics, CAM use, and HRQOL.

Part I: Sociodemographic and health-related characteristics (9 items), including gender, age, marital status, education, employment, monthly income, duration on hemodialysis, and comorbidities.

Part II: CAM-related information (15 items), including type and form of CAM used, sources of information and procurement, satisfaction, side effects, reasons for use or non-use, and disclosure to physicians. Participants were classified as CAM users if they had used at least one type of CAM more than three times within the previous six months. This threshold is adopted from the operational classification of frequent use of traditional Chinese medicine (TCM) in Taiwan’s National Health Insurance database [20,21], rather than a verified indicator of treatment intensity. The list of CAM modalities included in the questionnaire was developed based on the prior literature on CAM use among patients with MHD [18]. For conceptual purposes and drawing on the U.S. National Cancer Institute classification descriptions of complementary and alternative medicine, CAM was classified into five broad categories: biologically based practices (vitamins, dietary supplements, botanicals, specific foods), manipulative and body-based practices (massage, reflexology), mind–body therapies (yoga, tai chi, qigong, meditation, biofeedback, exercise), whole medical systems (acupuncture, homeopathy, naturopathy, Ayurveda, TCM), and energy therapies (reiki, therapeutic touch) [22].

Part III: HRQOL was assessed using the validated Chinese version of the Kidney Disease Quality of Life Short-Form Survey (KDQOL-SF v1.3) [23]. The questionnaire consists of 19 domains with a total of 80 questions and a 100-point scale categorized into 3 components: physical component summary (PCS), mental component summary (MCS), and kidney disease component summary (KDCS). PCS and MCS are the generic SF-36 instrument, and the KDCS is a kidney-disease-specific instrument that focuses on kidney disease-specific health concerns in patients with end-stage renal disease. In PCS, there are 21 items categorized into 4 domains: physical function (3 items), role limitations caused by physical health problems (4 items), pain (2 items), and general health perception (5 items). In the MCS, there are 14 questions categorized into 4 domains: emotional well-being (5 items), role limitations caused by emotional health problems (3 items), social function (2 items), and energy/fatigue-related issues (4 items). In the KDCS, there are 43 questions categorized into 11 domains: symptom problem (12 items), effects of kidney disease on daily life (8 items), burden of kidney disease (4 items), work status (2 items), cognitive function (3 items), quality of social interactions (3 items), sexual function (2 items), sleep (4 items), social support (2 questions), dialysis staff encouragement (2 items), and patient satisfaction (1 item). Scores for all subscales are transformed to a range between 0 and 100, with higher scores indicating better HRQOL [24]. The overall HRQOL is summarized into three composite scores: PCS (physical component summary), MCS (mental component summary), and KDCS (kidney disease component summary). The tool’s reliability has been previously confirmed, with Cronbach’s alpha ranging from 0.70 to 0.95 [25,26].

### 2.3. Data Collection

Following IRB approval, eligible patients were approached, informed about the study objectives, and asked for written consent. Questionnaires were completed through face-to-face interviews during dialysis sessions to minimize misinterpretation and missing data. Interviews were conducted by trained research assistants who were independent of the dialysis unit and had no prior relationship with the participants, thus avoiding potential conflicts of interest.

### 2.4. Statistical Analysis

Prior to data entry, completed questionnaires were verified for accuracy. Statistical analyses were performed using IBM SPSS Statistics for Windows, version 21.0 (IBM Corp., Armonk, NY, USA). Descriptive statistics (frequencies, percentages, and means ± standard deviations) were utilized to summarize baseline characteristics. KDQOL-SF v1.3 scores were derived based on the publicly available RAND scoring algorithm and linearly transformed to a 0–100 scale [24].

Differences between CAM users and non-users were evaluated using Pearson’s chi-square test for categorical variables. Student’s *t*-test was performed to compare continuous variables, including the 14 domain scores of the KDQOL-SF v1.3, between the two groups. Variables demonstrating a significance of *p* < 0.2 in the univariate analysis were subsequently entered into a multivariate logistic regression model, with adjusted odds ratios (AORs) and 95% confidence intervals (CIs) reported. To control for Type I error across the 14 domain-level comparisons (including symptoms and problems of kidney disease (SPKD), effects of kidney disease (EKD), burden of kidney disease (BKD), work status, cognitive function, quality of social interaction, social support, sexual function, sleep, dialysis staff encouragement, patient satisfaction, overall health score, PCS, and MCS), Bonferroni correction, consistent with the reported outcomes, was implemented, with statistical significance established at a threshold of *p* < 0.0036 (0.05/14). A general linear model was used to compare quality of life scores between CAM users and non-users, calculating mean differences after adjusting for age, gender, duration of dialysis, and comorbidities.

## 3. Results

### 3.1. Study Population Demographics of CAM Use

Of the 163 patients receiving MHD who were invited to participate, 154 completed the questionnaire and formed the final analysis, yielding a response rate of 94.5%. Nine nonrespondents with general survey fatigue and a poor general health condition were excluded from the analysis. The sociodemographic and clinical characteristics of the participants are summarized in Table 1. One-hundred and ten (71.4%) patients were aged 60 or above, eighty-seven (56.5%) were female, eighty (51.9%) were secondary graduates, one-hundred and thirty-five (87.7%) had married status, one-hundred and thirty-two (85.7%) were unemployed, and eleven (7.1%) had an average monthly income that resulted in them being considered well-off. One-hundred patients (64.9%) had been on dialysis for over 5 years, and the majority of patients (89%) had 1–3 co-existing conditions. Eighty-six patients (55.8%) reported having used CAM treatment. No significant differences were observed between CAM users and all of the population with respect to age, education, marital status, employment, monthly income, duration on hemodialysis, or comorbidities, with the exception of gender (*p* = 0.002).

### 3.2. Predictors of CAM Use

Gender was significantly associated with CAM use in the univariate analysis (OR = 2.79, 95% CI: 1.44–5.39, *p* = 0.002). After adjusting for the duration of dialysis, female sex remained an independent predictor of CAM use in the multivariable logistic regression model (AOR = 2.56, 95% CI: 1.28–5.13, *p* = 0.008), with females exhibiting 2.56 times higher odds of use than males (Table 2).

### 3.3. Characteristics and Type of CAM Use

**Figure 1:**
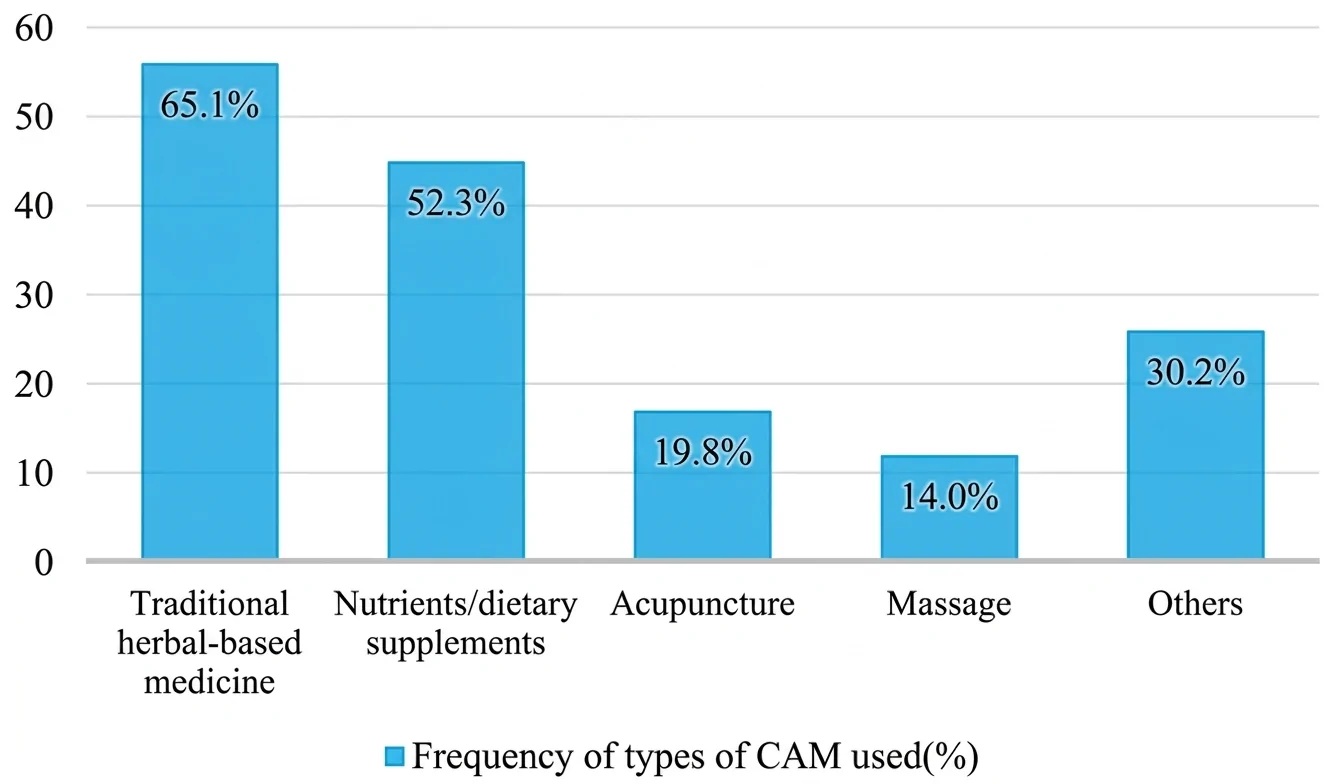
Frequency of different types of CAMs used by the study participants (*n* = 86).

The distribution of CAM modalities used is shown in Figure 1. The most frequently reported form was traditional herbal-based medicine, followed by nutrients/dietary supplements and acupuncture. Massage and others (qigong, yoga, essential oils, reflexology, etc.) ranked fourth and fifth in use, respectively.

The characteristics of CAM use are presented in Table 3. The most common source of CAM information was family members and friends (28, 32.6%), followed by other dialysis patients (26, 30.2%), and healthcare professionals (21, 24.4%). The leading reasons for CAM use were the perceived synergistic effect with conventional therapy (38, 44.2%), belief in CAM’s benefits (31, 36.0%), family tradition or cultural influence (23, 26.7%), and reduction in side effects from conventional therapy (19, 22.1%).

Among non-users (*n* = 68), the main reasons for not using CAM included lack of awareness (36.7%), disbelief in its benefits (23.5%), perceived additional burden (23.5%), and fear of side effects (19.1%).

Most CAM users (57, 66.3%) reported discussing their CAM use with physicians. Their top four reasons for discussion were as follows: I need to get the best medical advice from a physician (20, 35.1%), I am concerned about electrolyte (Ca, P, Na) overload (15, 26.3%), I worry about drug interactions (11, 19.3%), and I prefer to have a physician’s approval (8, 14.0%).

Among the 29 patients who reported not discussing CAM therapies with their physician, the most frequent reason was the belief that it was unnecessary for doctors to know (15, 51.7%), followed by the perception that consultation was not needed (14, 48.3%). Notably, none of the CAM users reported experiencing adverse effects. Furthermore, 90.7% (*n* = 78) expressed intent to continue CAM use, and only 2.3% reported dissatisfaction with CAM.

### 3.4. Association Between CAM Use and HRQOL

The mean scores for each KDQOL-SF v1.3 subscale are presented in Table 4. No significant differences were found between CAM users and non-users in most KDQOL-SF v1.3 domains. Nevertheless, CAM users exhibited significantly higher scores in terms of MCS (*p* = 0.001) and social support (*p* < 0.001) after Bonferroni adjustment was applied. Data on sexual function was not available due to the sensitive nature of the questions; all the participants declined to answer.

### 3.5. Adjusted Effect Size Analysis of HRQOL Differences Between CAM Users and Non-Users

For further analysis, a general linear model was used to estimate mean differences in quality of life scores between CAM users and non-users, adjusting for age, gender, duration of dialysis, and comorbidities. The results showed that CAM users had higher social support (mean difference: 9.06 ± 2.65; 95% confidence interval [CI]: 3.82 to 14.30; *p* = 0.001) and MCS (mean difference: 5.41 ± 1.68; 95% CI: 2.09 to 8.73; *p* = 0.002) scores compared to non-users. Conversely, the symptoms and problems of kidney disease (SPKD) scores in CAM users were slightly lower than in non-users (mean difference: −3.56 ± 1.58; 95% CI: −6.68 to −0.44; *p* = 0.026) (Table 5).

## 4. Discussion

As found in our previous study [18], the results revealed that about half of the patients used CAM, with traditional herbal-based medicine and nutrient/dietary supplements being the most commonly used treatments. The primary sources of information about CAM for patients were their family members/friends, and other dialysis patients, and the majority did not discuss CAM use with healthcare providers (HCPs). Female gender was identified as a predictor of CAM use. Regarding the positive influence of CAM on HRQOL, only the social support and mental component scores were found to be statistically significant.

In our present study, females were more likely than males to use CAM modalities. This finding is consistent with reports spanning several decades [27,28,29], which have shown higher CAM utilization among women. This trend may reflect broader gendered patterns of health behavior, as women seem to engage with health issues, seek help from HCPs, and visit physicians—including CAM practitioners—more frequently than men [30].

Although CAM use by MHD patients is extensively documented, research on patients’ perspectives regarding CAM use in dialysis settings remains extremely limited. Our study of 154 MHD patients found that 55.8% used CAM; however, only 66.3% discussed these therapies with their physicians, despite multiple opportunities for interaction during initial consultations and throughout their dialysis course. This low rate of communication with physicians is concerning. While some CAM therapies may be beneficial—such as acupuncture for uremic pruritus and muscle cramps [31,32], or herbal acupoint therapy for symptomatic intradialytic hypotension [33]—others are specifically contraindicated and can impair the effectiveness of hemodialysis [34].

The rate of patient–physician CAM communication in our study is comparable to the 63.2% reported among MHD patients in Trinidad [15] but higher than the 45.9% reported in South Korea [19]. Patients’ primary rationales for discussing CAM therapies with physicians—such as seeking optimal medical advice, addressing concerns over electrolyte overload and drug interactions, and seeking physician approval—have not been explored thoroughly in global CKD studies. This under-exploration may relate to the fact that dialysis patients often exhibit lower treatment compliance and adherence than other chronic disease patients due to the extreme complexity, lifestyle restrictions, and psychological burden of their regimen [35,36]. The disclosure rate observed in this study might be attributed to patients’ acceptance of their illness, fear of complications, peer encouragement, and continuous family and social support, which are particularly prominent within Taiwan’s interconnected society.

Among the patients in our study who did not disclose CAM use to their physicians, the most common reasons cited were perceptions that CAM use was “not important” or that there was “no need” to discuss it. These attitudes may stem from beliefs that CAM is natural, safe, culturally embedded, or intended for general well-being rather than disease treatment, making it seem unrelated to conventional medical care. However, for patients undergoing hemodialysis, taking unauthorized CHM, dietary nutrients, or over-the-counter supplements carries a high risk of life-threatening complications [37,38,39]. Because impaired kidneys cannot effectively filter active compounds, heavy metals, or mineral waste, these substances can easily build up to toxic levels, interfere with prescription medications, or severely destabilize electrolyte balance [34]. In additional, Kennedy et al. [40] reported that barriers to communication about CAM include perceived physician antagonism toward CAM and patients’ anticipation of discouraging responses. Poor communication between physicians and CAM-using patients may pose severe health risks due to potential toxicity or interactions with conventional treatments. Therefore, HCPs should proactively inquire about CAM use and foster open, nonjudgmental dialog to support safe and rational CAM practices.

In our study, the most common information sources about CAM were family members/friends (32.6%) and other dialysis patients (30.2%), whereas social media was the least frequently cited source (1.16%). This finding partially aligns with a study from Malaysia, which also identified family and relatives as the primary sources of CAM-related information [41]. Similar results were reported in a study conducted in Trinidad, where the predominant information sources were outside the medical community and included family members (68.4%), friends (42.1%), and other patients (42.1%) [15]. In contrast, several other studies have identified mass media, including the internet, television, radio, newspapers, and magazines, as the main sources of CAM information [15,42]. With the increasing popularity of social media, exaggerated claims regarding the efficacy of herbal products and dietary supplements—and their potential interference with evidence-based medical care—may become an increasingly serious concern [43]. Another point to note is that over 30% of CAM information comes from other dialysis patients. Dialysis patients often form a unique “dialysis support group” within the dialysis unit due to their long-term and frequent visits [44]. They face the shared illness together, easily exchanging information on lifestyle adjustments and emotional support. Previous research shows that peer-experience-driven choices significantly influence the adoption of CAM, guiding users to build trust, choose treatments, and gather information through social networks rather than informal medical channels [45,46]. However, this type of information carries many potential risks. For example, it can lead patients to ignore individual differences, discontinue medication on their own, refuse standard treatment, and even be misled by commercial groups [47].

Factors in the present study included variables related to potential disability, which may help examine the impact of social support and MCS on HRQOL, comparing CAM users with non-CAM users among MHD patients. CAM use was associated with selected psychosocial dimensions of HRQOL in this cohort, while no generalized improvement across all HRQOL domains can be inferred. Rather than being viewed solely as a means of addressing physical symptoms, CAM use may be embedded within patients’ broader approaches to coping with chronic illness and maintaining psychological and social well-being [48]. Certain CAM modalities [17], such as relaxation techniques, acupuncture, and qigong, may contribute to stress reduction, emotional regulation, and perceived psychological resilience through mind–body interactions and modulation of autonomic nervous system balance. In addition, individuals who actively seek CAM may perceive themselves to be taking a more active role in managing their health, which could be associated with greater self-efficacy and a stronger sense of personal agency.

The higher social support scores observed among CAM users further suggest that CAM use may be closely connected to interpersonal and social resources. In our study, family members, friends, and fellow patients were the primary sources of CAM information, indicating that CAM-related knowledge and experiences are often transmitted through patients’ existing social networks. The process of discussing CAM, seeking recommendations, and sharing treatment-related information can foster opportunities for interpersonal interaction and mutual support, a dynamic particularly relevant for patients undergoing long-term hemodialysis. Such social interactions may help mitigate feelings of isolation and reinforce patients’ perceived social resources. Conversely, patients with stronger social networks or greater perceived social support may also be more inclined to encounter, consider, and adopt CAM therapies [49]. Thus, the observed association between CAM use and social support may reflect a reciprocal relationship between CAM-related behaviors and patients’ social environments rather than a direct effect of CAM itself [50].

Taken together, the concurrent differences in MCS and social support suggest that the relationship between CAM use and HRQOL involves interconnected psychological and social dimensions. Psychological well-being and social connectedness likely reinforce one another: greater social support can facilitate emotional coping and reduce psychological distress, whereas better mental health enables patients to maintain interpersonal relationships and actively seek health-related resources. From a biopsychosocial perspective, CAM use may represent one component of a broader pattern of health-seeking behavior that encompasses psychological coping, social connectedness, and active engagement in chronic disease management. Importantly, our findings do not establish that CAM itself directly improves mental health or social support. Prospective longitudinal studies are warranted to determine how specific CAM modalities, patterns of engagement, or CAM-related social interactions contribute to subsequent changes in HRQOL, thereby clarifying the directionality of these associations.

While CAM users reported slightly lower SPKD scores (indicating a higher symptom burden), this association should be viewed through the lens of a demand-driven or self-selection mechanism. Our findings align with socio-behavioral models suggesting that patients with more severe clinical symptoms or perceived physical burdens are more inclined to seek CAM as a complementary coping strategy [51]. In addition, reverse causation remains a plausible explanation for these observed relationships. For instance, lower SPKD scores might reflect baseline disease severity driving CAM utilization, rather than a negative impact of CAM on physical health. However, given the cross-sectional nature of the present study, these potential explanations should be interpreted as hypotheses rather than proven causal mechanisms.

Based on our empirical findings, we propose the following concrete clinical and policy actions: (1) Given that 33.7% of users do not disclose CAM use because they deem it unimportant or unnecessary, HCPs should consider moving beyond voluntary disclosure and routinely asking patients about CAM use. Assessment of CAM use in dialysis care may help identify potential herb–drug interactions or toxicities. (2) Because female sex was independently associated with CAM use, HCPs should consider sex-related differences in CAM utilization when assessing patients’ complementary therapy practices while ensuring that all patients receive consistent, nonjudgmental counseling. (3) Since other patients (30.2%) and family/friends (32.6%) are the dominant information sources, hospitals should develop peer-mentor training programs. By educating influential lead patients on the evidence-based safety profiles of common CAMs, healthcare providers can leverage existing social networks to disseminate accurate health information more effectively than traditional top-down physician education.

Several limitations should be acknowledged when interpreting the results of this study. First, as the investigation was conducted in a single dialysis center, the findings on CAM use may introduce selection bias and reduce the generalizability of all MHD patients in Taiwan. Second, due to the cross-sectional design, causal relationships between CAM use and the observed outcomes cannot be established. Longitudinal studies are necessary to provide a more comprehensive understanding of the factors influencing HRQOL among hemodialysis patients. Third, selection bias might have been introduced due to the characteristics of the nonrespondents not being available, although the response rate was high. Fourth, although the KDQOL-SF v1.3 is a valuable tool for assessing life quality across different stages of kidney disease, it has some limitations, such as a high burden, low reliability in the subscales of work status and quality of social interaction, potential ceiling effects, and limited adaptability to evolving healthcare contexts. Moreover, some patients have reported that it does not fully capture all aspects of their well-being [52,53]. Fifth, it is possible that certain non-CAM users had in fact used CAM fewer than the minimum defined frequency (three times), which may have led to an underestimation of CAM prevalence. Finally, we emphasize that CAM encompasses heterogeneous practices, including herbal products, nutritional supplements, acupuncture, massage, and mind–body practices. These modalities differ in their mechanisms, frequency of use, indications, and potential clinical risks. Therefore, the binary CAM-use variable should not be interpreted as homogeneous therapeutic exposure, and the observed associations cannot be attributed to any specific modality.

## 5. Conclusions

In this cross-sectional study of patients receiving MHD, female sex was independently associated with CAM use. Although most CAM users reported discussing their CAM use with physicians, approximately one-third did not, highlighting the need for proactive, open, and nonjudgmental communication about complementary therapies in routine dialysis care. CAM use was also associated with higher mental component summary scores and perceived social support, whereas most other health-related quality-of-life domains did not differ significantly between CAM users and non-users. These findings suggest that CAM use may be linked to patients’ psychosocial characteristics and health-seeking behaviors rather than reflecting a direct therapeutic effect. HCPs should routinely assess CAM use, provide individualized safety counseling, and consider patients’ psychosocial needs when discussing complementary therapies. Further multicenter longitudinal studies are warranted to clarify the direction of these associations and their implications for long-term health-related quality of life and clinical outcomes.

## Figures and Tables

**Table 1:** Sociodemographic and clinical characteristics of MHD patients (*n* = 154).

Characteristics	No. of Participants (% of Total)	No. of Patients Who Have Used CAM (%)	*p*-Value ^†^
Total	154 (100%)	86 (55.8)	
Age (year)			0.364
<40	5 (3.2)	4 (80)	--
41–60	39 (25.3)	24 (61.5)	--
61–80	110 (71.4)	58 (52.7)	--
Gender			0.002
Male	67 (43.5)	28 (41.8)	--
Female	87 (56.5)	58 (66.7)	--
Education status			0.539
Primary	56 (36.4)	29 (51.8)	--
Secondary	80 (51.9)	45 (56.3)	--
Tertiary	18 (11.7)	12 (66.7)	--
Marital status			0.493
Single	19 (12.3)	12 (63.2)	--
Ever married	135 (87.7)	74 (54.8)	--
Employment status			0.74
Unemployed	132 (85.7)	73 (55.3)	--
Employed	22 (14.3)	13 (59.1)	--
Average monthly income (US dollars)			0.756
<930	143 (92.9)	79 (55.2)	--
≥930	11 (7.1)	7 (63.6)	--
Duration of dialysis			0.121
<1 year	20 (13)	7 (35)	--
1–5 year	34 (22.1)	19 (55.9)	--
>5 year	100 (64.9)	60 (60)	--
Comorbidity			0.854
No.	10 (6.5)	6 (60)	--
1–3	137 (89)	77 (56.2)	--
>3	7 (4.5)	3 (42.9)	--

^†^ χ^2^ test. CAM: complementary and alternative medicine; MHD: maintenance hemodialysis.

**Table 2:** Factors associated with patients who have used CAM.

Demographic and Clinical Variables	Univariate Analysis		Multivariate Logistic Analysis	
	OR (95% CI)	*p*-Value	AOR (95% CI)	*p*-Value
Age				
<40	1.00 (ref)	0.368		
41–60	0.4 (0.041–3.927)	0.432		
61–80	0.279 (0.03–2.575)	0.260		
Gender				
Male	1.00 (ref)			
Female	2.786 (1.441–5.385)	0.002	2.558 (1.276–5.131)	0.008
Education				
Primary	1.00 (ref)	0.544		
Secondary	1.197 (0.603–2.376)	0.607		
Tertiary	1.862 (0.613–5.658)	0.273		
Marital status				
Single	1.00 (ref)			
Ever married	0.708 (0.262–1.908)	0.494		
Employment				
Unemployed	1.00 (ref)			
Employed	1.167 (0.467–2.919)	0.741		
Average monthly income (US dollars)				
<930	1.00 (ref)			
≥930	1.418 (0.397–5.058)	0.591		
Duration of dialysis				
<1 year	1.00 (ref)	0.134		0.418
1–5 year	2.352 (0.751–7.365)	0.142	2.093 (0.65–6.736)	0.215
>5 year	2.786 (1.023–7.589)	0.045	1.924 (0.67–5.529)	0.224
Comorbidity				
No	1.00 (ref)	0.761		
1–3	0.856 (0.231–3.169)	0.815		
>3	0.5 (0.070–3.55)	0.488		

CAM: complementary and alternative medicine.

**Table 3:** Characteristics of CAM use in the study population (*n* = 154).

Variables About CAM Use	Frequency (%)
CAM use (*n* = 154)	
Yes	55.8%
No	44.2%
Source of information about CAM (*n* = 86)	
Family members and friends	32.6%
Other dialysis patients	30.2%
Healthcare professionals	24.4%
Traditional media (television, radio, and newspaper)	12.8%
Others (threads, TikTok, Facebook)	1.16%
Reasons for CAM use (*n* = 86)	
Synergic effect of conventional therapy	44.2%
Belief in advantages of CAM	36.0%
Family tradition/culture	26.7%
Decrease in side effects of conventional therapy	22.1%
Dissatisfaction with conventional therapy	4.7%
Emotional support	2.3%
Boosting immune system	0
Treatment of other medical problems	0
Others	0
Reasons for not using CAM among non-users (*n* = 68)	
Never heard of CAM	36.7%
Lack of belief in the benefits of CAM	23.5%
Additional burden	23.5%
Afraid of side effects	19.1%
The doctor did not advise CAM	7.4%
Consult doctor about CAM use (*n* = 86)	
No	33.7%
Yes	66.3%
Reason for consulting a physician (*n* = 57 )	
Need the best medical advice from a physician	35.1%
Avoid electrolyte (Ca, P, Na) overload	26.3%
Concern about drug interactions	19.3%
Prefer to have a physician’s approval	14.0%
Others	5.3%
Reason for not consulting a physician (*n* = 29)	
It was not important for doctor to know about my CAM use	51.7%
No need to consult doctor	48.3%
Insufficient information about CAM	0
Anticipating negative response about CAM use	0
Others	0
Side effects from CAM (*n* = 86)	
No	100%
Yes	0
Satisfaction with CAM (*n* = 86)	
Satisfied	72.1%
Neutral	25.6%
Dissatisfied	2.3%
Would you use CAM again? (*n* = 86)	
No	9.3%
Yes	90.7%

**Table 4:** Quality of life (KDQOL-SF v1.3) scores among CAM users and non-CAM users (*n* = 154).

KDQOL-SF v1.3	Non-CAM Users (Mean ± SD)	CAM Users (Mean ± SD)	*p*-Value
Kidney disease component summary (KDCS)			
Symptom/problem list (SPKD)	87.3 ± 10.3	83.3 ± 8.3	0.009
Effects of kidney disease (EKD)	74.8 ± 16.1	76.5 ± 16	0.531
Burden of kidney disease (BKD)	55.4 ± 26.8	51.1 ± 22	0.284
Work status	44.1 ± 26.7	38.4 ± 30.4	0.221
Cognitive function	89.3 ± 16.1	89.3 ± 14.7	0.996
Quality of social interaction	89.4 ± 17.3	87.6 ± 16.1	0.503
Social support	73.8 ± 14.8	83.9 ± 16.3	<0.001 *
Sexual function	N/A	N/A	
Sleep	64.8 ± 25.2	67.7 ± 21.7	0.445
Dialysis staff encouragement	63.2 ± 14	65.8 ± 13	0.232
Patient satisfaction	68.6 ± 14	66.5 ± 6.5	0.243
Overall health score	58.2 ± 17.4	55.9 ± 18.8	0.437
36-item health survey (SF-36)			
SF12-Physical composite summary (PCS)	38.6 ± 9.2	39.1 ± 9.0	0.725
SF12-Mental composite summary (MCS)	47.1 ± 10.2	52.6 ± 10.2	0.001 *

* After Bonferroni correction for 14 comparisons, the significance threshold is α = 0.0036. N/A: not applicable. PCSs: physical component summaries; MCSs: mental component summaries; KDCSs: kidney-disease component summaries.

**Table 5:** Adjusted mean differences in quality of life (KDQOL-SF v1.3) scores between CAM users and non-users.

KDQOL-SF v1.3	Mean Difference (Mean ± SE) * (CAM Users vs. Non-CAM Users)	95% CI	*p*-Value
Kidney disease component summary (KDCS)			
Symptom/problem list (SPKD)	−3.56 ± 1.58	(−6.68, −0.44)	0.026
Effects of kidney disease (EKD)	2.17 ± 2.68	(−3.12, 7.47)	0.418
Burden of kidney disease (BKD)	−3.47 ± 4.05	(−11.47, 4.53)	0.392
Work status	−3.71 ± 4.79	(−13.18, 5.76)	0.44
Cognitive function	0.06 ± 2.48	(−4.84, 4.95)	0.982
Quality of social interaction	−0.79 ± 2.80	(−6.33, 4.74)	0.778
Social support	9.06 ± 2.65	(3.82, 14.3)	0.001
Sexual function	N/A	N/A	
Sleep	2.13 ± 3.94	(−5.65, 9.91)	0.59
Dialysis staff encouragement	2.2 ± 2.27	(−2.3, 6.68)	0.335
Patient satisfaction	−1.641 ± 1.792	(−5.183, 1.9)	0.361
Overall health score	−1.98 ± 3.1	(−8.10, 4.15)	0.525
36-item health survey (SF-36)			
SF12-Physical composite summary (PCS)	0.11 ± 1.52	(−2.89, 3.10)	0.945
SF12-Mental composite summary (MCS)	5.41 ± 1.68	(2.09, 8.73)	0.002

* After Bonferroni correction for 14 comparisons, the significance threshold is α = 0.0036. N/A: not applicable. PCSs: physical component summaries; MCSs: mental component summaries; KDCSs: kidney-disease component summaries.

## Data Availability

The data are not publicly available due to privacy and ethical restrictions. De-identified data may be available from the corresponding author upon reasonable request, subject to applicable ethical and privacy requirements.

## Abbreviations

The following abbreviations are used in this manuscript:

CAM	complementary and alternative medicine
CKD	chronic kidney disease
HRQOL	health-related quality of life
HD	hemodialysis
HCPs	healthcare providers
ESRD	end-stage renal disease
WHO	World Health Organization
MHD	maintenance hemodialysis
TCM	traditional Chinese medicine
KDQOL	kidney disease quality of life
PCS	physical component summary
MCS	mental component summary
BKD	burden of kidney disease
SPKD	symptoms and problems of kidney disease
EKD	effects of kidney disease
IRB	institutional review board
ORs	odds ratios
CIs	confidence intervals
KDCS	kidney-disease component summaries.

## Appendix A

**Table A1:** STROBE Statement—checklist of items that should be included in reports of observational studies.

	Item No.	Recommendation	Page No.	Relevant Text from Manuscript
Title and abstract	1	(a) Indicate the study’s design with a commonly used term in the title or the abstract	1	Title: “Complementary and Alternative Medicine Use and Its Association with Health-Related Quality of Life in Patients Receiving Maintenance Hemodialysis: A Cross-Sectional Study.”
		(b) Provide in the abstract an informative and balanced summary of what was done and what was found	2	Abstract: “This single-center cross-sectional study included 154 patients receiving MHD in southern Taiwan.” The abstract summarizes CAM use, analyses, key results, and conclusions.
Introduction				
Background/rationale	2	Explain the scientific background and rationale for the investigation being reported	3–4	Introduction, lines 63–110: background on ESRD/MHD, HRQOL burden, CAM use among dialysis patients, and the evidence gap motivating this study.
Objectives	3	State specific objectives, including any prespecified hypotheses	4	Introduction, lines 104–111: “aimed to investigate the predictors of CAM use, the impact on patients’ HRQOL, and to gain a comprehensive understanding of patients’ perspectives of CAM use.”
Methods				
Study design	4	Present key elements of study design early in the paper	4	Methods, Section 2.1: “From August 2022 to July 2024…”; single-center cross-sectional study of patients receiving MHD.
Setting	5	Describe the setting, locations, and relevant dates, including periods of recruitment, exposure, follow-up, and data collection	4	Section 2.1: “From August 2022 to July 2024” at the dialysis center of Chang Gung Memorial Hospital, Kaohsiung branch, Taiwan.
Participants	6	(a) Cohort study—Give the eligibility criteria, and the sources and methods of selection of participants. Describe methods of follow-up Case–control study—Give the eligibility criteria, and the sources and methods of case ascertainment and control selection. Give the rationale for the choice of cases and controls Cross-sectional study—Give the eligibility criteria, and the sources and methods of selection of participants	4	Section 2.1: eligibility included ESRD patients receiving MHD for >3 months; exclusions were age <20 years, inability to speak Mandarin or Hokkien, dementia, or psychiatric illness. A random sample was selected from the dialysis registration list.
		(b) Cohort study—For matched studies, give matching criteria and number of exposed and unexposed Case–control study—For matched studies, give matching criteria and the number of controls per case	N/A	Not applicable: this is a cross-sectional study; no matching of exposed and unexposed groups was used.
Variables	7	Clearly define all outcomes, exposures, predictors, potential confounders, and effect modifiers. Give diagnostic criteria, if applicable	4–5	Section 2.1, Section 2.2, Section 2.3 and Section 2.4 define CAM use (>3 uses in the previous 6 months), HRQOL measured by KDQOL-SF v1.3, candidate predictors, and adjustment variables (age, gender, dialysis duration, and comorbidities).
Data sources/measurement	8 *	For each variable of interest, give sources of data and details of methods of assessment (measurement). Describe comparability of assessment methods if there is more than one group	4–5	Section 2.2, Section 2.3 and Section 2.4: structured questionnaire covering demographics, CAM use, and HRQOL; validated Chinese KDQOL-SF v1.3; RAND scoring algorithm with scores transformed to 0–100.
Bias	9	Describe any efforts to address potential sources of bias	5, 15	Methods: face-to-face interviews by trained research assistants independent of the dialysis unit to minimize misinterpretation and missing data. Limitations: potential selection bias and lack of nonrespondent characteristics are acknowledged.
Study size	10	Explain how the study size was arrived at	4	Section 2.1: sample size was calculated as 128 using G*Power 3.1.9.2, with target increased to 154 to reduce nonresponse and improve reliability.
Quantitative variables	11	Explain how quantitative variables were handled in the analyses. If applicable, describe which groupings were chosen and why	4–5	CAM use was categorized as >3 uses in the previous 6 months, based on the operational classification of frequent TCM use in Taiwan’s National Health Insurance database. Continuous outcomes were summarized as means and SDs; KDQOL-SF scores were transformed to 0–100.
Statistical methods	12	(a) Describe all statistical methods, including those used to control for confounding	5–6	Section 2.4: descriptive statistics, chi-square tests, Student’s *t*-tests, multivariable logistic regression with AORs and 95% CIs, Bonferroni correction (*p* < 0.0036), and general linear models adjusted for age, gender, dialysis duration, and comorbidities.
		(b) Describe any methods used to examine subgroups and interactions	Not reported	No subgroup or interaction analyses are described in the Methods or Results.
		(c) Explain how missing data were addressed	5, 10	Section 2.2 reports that all participants declined the sexual-function questions; Results, Section 3.3, states sexual-function data were unavailable for this reason. Handling of other missing data is not explicitly described.
		(d) Cohort study—If applicable, explain how loss to follow-up was addressed Case–control study—If applicable, explain how matching of cases and controls was addressed Cross-sectional study—If applicable, describe analytical methods taking account of sampling strategy	4–5	Section 2.1 describes simple random sampling from the dialysis registration list; Section 2.4 describes the statistical methods. No loss-to-follow-up applies to this cross-sectional study.
		(e) Describe any sensitivity analyses	Not reported	No sensitivity analyses are reported.
Results				
Participants	13 *	(a) Report numbers of individuals at each stage of study—eg numbers potentially eligible, examined for eligibility, confirmed eligible, included in the study, completing follow-up, and analyzed	6	Results, Section 3.1: 163 patients were invited, 154 completed the questionnaire and were analyzed (response rate 94.5%).
		(b) Give reasons for non-participation at each stage	6	Results, Section 3.1: “Nine nonrespondents with general survey fatigue and poor general health condition were excluded from the analysis.”
		(c) Consider use of a flow diagram	6	Participant numbers and reasons for nonparticipation are reported in text; no participant flow diagram is presented.
Descriptive data	14 *	(a) Give characteristics of study participants (e.g., demographic, clinical, social) and information on exposures and potential confounders	6–8	Table 1 reports sociodemographic and clinical characteristics. Table 3 reports CAM use, information sources, reasons for use/non-use, physician disclosure, reported side effects, satisfaction, and intention to continue.
		(b) Indicate number of participants with missing data for each variable of interest	10	Results, Section 3.3: sexual-function data were unavailable because all participants declined to answer; numbers of missing data for other variables are not separately reported.
		(c) Cohort study—Summarize follow-up time (e.g., average and total amount)	N/A	Not applicable to this cross-sectional study.
Outcome data	15 *	Cohort study—Report numbers of outcome events or summary measures over time	N/A	Not applicable: no cohort follow-up or outcome events over time.
		Case–control study—Report numbers in each exposure category, or summary measures of exposure	8–10	Table 3 reports exposure-category summaries: 86/154 participants (55.8%) were CAM users and 68/154 (44.2%) were non-users; CAM modalities and related characteristics are described.
		Cross-sectional study—Report numbers of outcome events or summary measures	10–11	Table 4 reports unadjusted KDQOL-SF domain and summary scores by CAM-use group; Table 5 reports adjusted mean differences, standard errors, 95% CIs, and *p*-values.
Main results	16	(a) Give unadjusted estimates and, if applicable, confounder-adjusted estimates and their precision (e.g., 95% confidence interval). Make clear which confounders were adjusted for and why they were included	8, 11	Table 2 reports unadjusted ORs and adjusted ORs with 95% CIs for CAM use. Table 5 reports adjusted HRQOL mean differences and 95% CIs; models adjusted for age, gender, dialysis duration, and comorbidities.
		(b) Report category boundaries when continuous variables were categorized	4, 6–8	Section 2.2 defines CAM users as those reporting use more than three times in the previous six months. Table 1 presents categorized age, dialysis duration, income, and comorbidity variables.
		(c) If relevant, consider translating estimates of relative risk into absolute risk for a meaningful time period	N/A	Not applicable: the cross-sectional study reports prevalence and associations rather than relative risks over a defined follow-up period.
Other analyses	17	Report other analyses done—e.g., analyses of subgroups and interactions, and sensitivity analyses	8–11	Other analyses include multivariable logistic regression for predictors of CAM use and general linear models for adjusted HRQOL mean differences (Table 2 and Table 5). No subgroup, interaction, or sensitivity analyses are reported.
Discussion				
Key results	18	Summarize key results with reference to study objectives	11–15	Discussion and Conclusions summarize the principal findings: CAM use prevalence, female sex as a predictor, physician disclosure patterns, and associations with MCS and social support.
Limitations	19	Discuss limitations of the study, taking into account sources of potential bias or imprecision. Discuss both direction and magnitude of any potential bias	14–15	Limitations paragraph: single-center design, cross-sectional design and inability to infer causality, possible selection bias due to unavailable nonrespondent characteristics, KDQOL-SF limitations, possible underestimation of CAM prevalence, and heterogeneous CAM modalities.
Interpretation	20	Give a cautious overall interpretation of results considering objectives, limitations, multiplicity of analyses, results from similar studies, and other relevant evidence	13–15	Discussion interprets the MCS/social-support findings cautiously and notes that associations may reflect reciprocal psychosocial relationships; it states that causal relationships cannot be established and further longitudinal studies are warranted.
Generalizability	21	Discuss the generalizability (external validity) of the study results	14–15	Limitations: the single-center sample may reduce generalizability to all MHD patients in Taiwan; the Conclusions call for multicenter longitudinal studies.
Other information				
Funding	22	Give the source of funding and the role of the funders for the present study and, if applicable, for the original study on which the present article is based	15	Funding statement lists support from Kaohsiung Chang Gung Memorial Hospital, Taiwan Ministry of Health and Welfare, China Medical University, and the National Science and Technology Council. The funders’ roles are not explicitly stated in the visible funding statement.

* Give information separately for cases and controls in case–control studies and, if applicable, for exposed and unexposed groups in cohort and cross-sectional studies. Note: An Explanation and Elaboration article discusses each checklist item and gives methodological background and published examples of transparent reporting. The STROBE checklist is best used in conjunction with this article (freely available on the Web sites of PLoS Medicine at http://www.plosmedicine.org/, Annals of Internal Medicine at http://www.annals.org/, and Epidemiology at http://www.epidem.com/). Information on the STROBE Initiative is available at www.strobe-statement.org(accessed on 15 September 2026).
